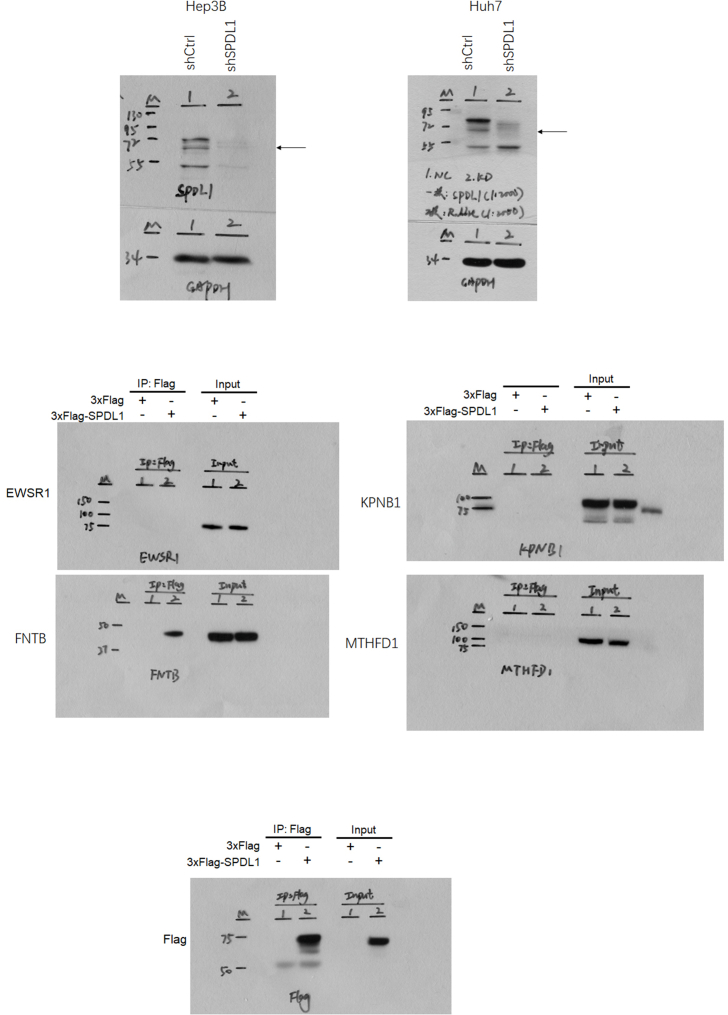# Silencing of spindle apparatus coiled-coil protein 1 suppressed the progression of hepatocellular carcinoma through farnesyltransferase-beta and increased drug sensitivity

**DOI:** 10.1016/j.heliyon.2024.e34484

**Published:** 2024-07-14

**Authors:** Yirui Zhai, Fan Wu, Xin Xu, Pan Zhao, Lingxia Xin, Mengyuan Li, Yuan Zong, Zhuanbo Yang, Zhuoran Li, Liming Wang, Bo Chen

**Affiliations:** a Department of Radiation Oncology, National Cancer Center/National Clinical Research Center for Cancer/Cancer Hospital, Chinese Academy of Medical Sciences and Peking Union Medical College, Beijing, 100021, China; b Department of Hepatobiliary Surgery, National Cancer Center/National Clinical Research Center for Cancer/Cancer Hospital, Chinese Academy of Medical Sciences and Peking Union Medical College, Beijing, 100021, China

**Keywords:** SPDL1, FNTB, Immunoprecipitation–mass spectrometry, High-content screening assay, Migration, Cell sensitivity

## Abstract

Hepatocellular carcinoma (HCC) is the major cause of cancer-associated mortality worldwide. Despite great advances have been made on the treatment of HCC, the survival rate of patients remains poor. Spindle apparatus coiled-coil protein 1 (SPDL1) is involved in the development of various cancers in humans. However, the role of SPDL1 in HCC remains unclear. In this study, we found high expression of SPDL1 in HCC tissues as compared to normal samples. *In vitro*, silencing of SPDL1 induced HCC cell apoptosis, and suppressed HCC cell propagation and migration. *In vivo*, knockdown of SPDL1 inhibited the tumor growth of HCC cells. These findings indicated the tumor-promoting role of SPDL1 in HCC. Mechanistically, we identified farnesyltransferase-beta (FNTB) as the downstream target protein of SPDL1 based on immunoprecipitation and mass spectrometry, which were confirmed by western blotting. Rescue assay determined that FNTB played a tumor promoting role in SPDL1-trigger HCC cell growth. Overexpression of FNTB recovered HCC cell viability and migration in SPDL1 knockdown cells. We also found that silencing of SPDL1 increased the sensitivity of Huh7 cells to sorafenib and lenvatinib, suggesting that SPDL1 is a new therapeutic target in HCC. Collectivity, the present study identified a new axis SPDL1/FNTB involved in the progression of HCC. Hence, SPDL1/FNTB is a potential target for the treatment of HCC.

## 1 Introduction

Hepatocellular carcinoma (HCC) is the sixth most common malignancy and the third leading cause of cancer-related mortality worldwide [1]. The World Health Organization estimated that >1 million individuals will expire due to liver cancer by 2030 [2]. HCC is distinct because its prognosis relies on tumor stage and disease severity [3]. The standard therapeutic approaches of HCC include radiotherapy, *trans*-arterial embolization, surgical resection, and chemotherapy. Nevertheless, delays in diagnosis often results in advanced HCC [4]. The majority of HCC patients diagnosed at late stage can only obtain limited durable response from the current therapies [5]. Hence, it is important to understand the pathophysiological mechanisms of HCC carcinogenesis. Discovering new diagnostic and treatment methods can improve the overall prognosis of patients with HCC.

Spindle apparatus coiled-coil protein 1 (SPDL1), which encodes Spindly, was discovered in 2007 [6]. It contains a coiled-coil domain and is currently recognized as a significant determining factor of DNA fidelity by ensuring faithful mitosis [7]. Spindly exerts a significant effect on a subset of functions of dynein/dynactin in cell migration [8]. Depletion of Spindly by RNA interference leads to widespread chromosome mis-segregation [9]. Recent studies have indicated that high expression of SPDL1 participates in the progression of human cancers. Human SPDL1 has been involved in proinflammatory reactions in patients with prostate cancer [10]. Chromosome misalignment and accumulation of lung cancer cells in mitosis were induced by suppression of Spindly through small-interfering RNA. Reduction of Spindly conferred these cells more sensitive to low-dose paclitaxel [11]. In addition, SPDL1 overexpression correlated with the poor prognosis of the patients with different malignancies, such as colorectal cancer (CRC) [7], pancreatic ductal adenocarcinoma [12,13], oral squamous cell carcinoma (OSCC) [14], lung cancer [[15], [16], [17]], and esophageal cancer (ESCA) [18]. However, the significance of SPDL1 in HCC remains unknown. Verifying the clinical relevance and biological function of SPDL1 in HCC carcinogenesis will help us to gain novel insight into the diagnosis and therapy for the patients.

The aim of this study was to investigate the function of SPDL1 in the development of HCC and its effect on drug sensitivity.

## 2 Materials and methods

### 2.1 The Cancer Genome Atlas (TCGA) samples

The expression of SPDL1 in patients with HCC was evaluated using TCGA data.

### 2.2 Cell culture and cell transfection

The Cell Bank of the Chinese Academy of Sciences (Shanghai, China) provided the human HCC cell lines Hep3B and Huh7. The cells were cultured in Dulbecco's altered Eagle medium supplemented with 10 % fetal bovine serum and 1 % antibiotics at 37 °C in a 5 % CO_2_ air atmosphere.

The lentiviral vector with SPDL1 short hairpin RNA (shRNA) plasmid, empty vector, FNTB overexpression vector plasmid, and empty vector plasmid were obtained from GeneChem (Shanghai, China). Lipofectamine 2000 was used for cell transfection. Polybrene was added to the medium to enhance the transduction of lentiviral particles. Reverse transcription-quantitative polymerase chain reaction (RT-qPCR) and western blotting were utilized to determine knockdown efficiency at 3 days after transduction.

### 2.3 RNA extraction and RT-PCR

The Qiagen RNeasy Plus Mini Kit was used to extract total RNA from cultured cells. Reverse transcription was performed with 1 μg of total RNA. The amplification of cDNA was conducted through SYBR green-based RT-qPCR on the CFX96 real-time PCR system (Bio-Rad, USA). Glyceraldehyde-3-phosphate dehydrogenase (GAPDH) was used as the internal control. The 2−ΔΔCt approach was used to calculate the relative expression [19]. The sequences of the primers were listed as follow: SPDL1, forward, 5′-AGGTAGACCGGCTTAAAGAGG-3’; reverse, 5′-ACGAGCTTTCTCTAGGGCATTAT-3’. GAPDH, forward, 5′-AATGGACAACTGGTCGTGGAC-3’; reverse, 5′-CCCTCCAGGGGATCTGTTTG-3’.

### 2.4 Western blotting analysis

RIPA assay buffer containing protease inhibitor was used to prepare whole cell lysates. The Bradford approach was adopted to measure the protein concentration. The protein lysates were loaded into prefabricate 10 % sodium dodecyl sulfate/polyacrylamide gel electrophoresis (SDS/PAGE) gels (Bio-Rad) for isolation, which was performed under 120 V for 1 h. Thereafter, the isolated proteins were transferred onto polyvinylidene difluoride membrane through the quick semidry method (Bio-Rad). Membranes were incubated at 4 °C overnight with primary antibodies against SPDL1 (1:1000; A301-354 A; Bethyl Laboratories), Ewing sarcoma breakpoint region 1 (EWSR1; 1:1000; PA5-29905, Thermo Fisher Scientific), FNTB (1:1000; PA5-97757; Thermo Fisher Scientific), karyopherin subunit beta 1 (KPNB1; 1:1000; A300-482 A; Bethyl Laboratories), and methylenetetrahydrofolate dehydrogenase, cyclohydrolase and formyltetrahydrofolate synthetase 1 (MTHFD1; 1:1000; A305-285 A; Bethyl Laboratories). An antibody against GAPDH (MA5-15738; Invitrogen) was used for the loading control.

### 2.5 Xenograft mouse model and small animal live imaging

Female BALB/C nude mice (5-week-old, Beijing Vital River Laboratory Animal Technology Co., Ltd.) were randomly separated into two groups (n = 10, per group). Huh7-control-shRNA-Red-fLuc and Huh7-SPDl1-shRNA-Red-fLuc cells (3 × 10^6^) were injected subcutaneously into the right flanks of the mice, respectively. The tumor volume was measured every two days and calculated using the formula length × width^2^ × 0.5 (mm^3^). The tumor size, volume and weight were measured when the mice were sacrificed at day 38 post-implantation.

When tumor diameter reached up to 1 cm, mice were analyzed on an IVIS spectrum optical imaging system (PerkinElmer, Lumina LT) every 2 days for a total of six times. The xenograft-bearing mice were injected with 15 mg/mL D-Luciferin (per 10 μl/g) via intraperitoneal. After 15 min, the mice were anesthesia with isoflurane gas using the built-in gas anesthesia system of the imaging system. The fluorescence was observed under the imaging device several minutes later. Animal operations were performed according to the National Institutes of Health guidelines, and approved by the Institutional Animal Care and Use Committee of Chinese Academy of Medical Sciences and Peking Union Medical College (NCC2022A481).

### 2.6 Cell apoptosis

Cell apoptosis was determined with an Annexin-V-APC kit (Ebioscience, United States). Briefly, after washing with PBS, the transfected cells were resuspended in staining buffer. Annexin V-APC reagent (5 μL) was added to cell suspension (100 μL), and the mixture was maintained at room temperature for 15 min. Thereafter, cell apoptosis was detected on flow cytometry (FACSCalibur, Becton-Dickinson, United States).

### 2.7 Cell proliferation

The measurement of cell propagation was conducted with the 3-(4,5-dimethylthiazol-2-Yl)-2,5-diphenyltetrazolium bromide (MTT) assay. After seeding equal number of shCtrl and shSPDL1 HCC cells, or treating the cells with the indicated drugs, such as sorafenib (BAY43–9006, Selleck, Shanghai, China) or lenvatinib (E7080, Selleck), cells were seeded into 96-well plates (2 × 10^3^/well). After 24 h, MTT (Yeasen, China) (10 μL) was added, and the cells were incubated for 4 h. Following the removal of supernatants, dimethyl sulfoxide (Yeasen) (150 μL) was added to the cells in darkness. The absorbance at 490 nm wavelength was measured for 1–4 days to determine the numbers of cells per well.

Cell viability was detected by the high-content screening (HCS) assay. Transfected cells were seeded in 96-well plates and cultivated for 1–5 days. Images were captured with a 20 × objective fluorescence-imaging microscope and analyzed with ArrayScan HCS software (Cellomics Inc.) [20].

### 2.8 Cell migration

Cell migration was tested with the Transwell assay. After transfection with shRNA for 3 days, the cells were trypsin and counted. A total of 1 × 10^5^ cells resuspended in serum-free medium were seeded into the upper chamber of a 24-well Transwell plate, while the complete medium including 10 % FBS was used to fill the lower chamber. Approximately 24 h later, migrated cells were detached from the bottom of the membrane. Detached cells were stained with Giemsa and counted under a light microscope.

Wound-healing assay was also utilized to measure cell migration. Transfected cells were seeded in a 6-well plate at 1 × 10^6^ cells per well and cultured overnight. Subsequently, a wound was developed with a sterile 200 μL pipette tip, and the cells were washed twice with PBS. Thereafter, the cells were cultured in serum-free medium for 48 h. The cells were photographed at various time points (i.e., 0 and 48 h) with a microscope to evaluate the migration distance.

### 2.9 Immunoprecipitation (IP) and mass spectrometry (MS)

The lysis of Huh7 cells was carried out with IP buffer on ice. Cells were sonicated and the lysates were centrifuged. The supernatant was incubated with *anti*-SPDL1 antibody and protein A/G beads (Santa Cruz, sc-2003) at 4 °C overnight on a rotating wheel. IP buffer was used to wash the immunoprecipitants. Next, SDS loading buffer was added, and proteins were eluted by boiling at 95 °C for 5 min.

For mass spectrometry assay, lysates from 293 T cells transfected with Flag-con or Flag-SPDL1 were centrifuged to remove cell debris. The lysates were subjected to IP with Flag M2 beads at 4 °C overnight. Bound proteins were eluted by boiling, resolved by SDS-PAGE and stained with Coomassie blue stain, followed by MS analysis on an ABI 4700 MALDI TOF.

### 2.10 Statistical analysis

Prism 8.0 (GraphPad) and SPSS 26.0 software (IBM Corporation, Armonk, NY, USA) packages were used for statistical analysis. Data represent the mean ± standard deviation. The normality assessment of the data was carried out. Student's *t*-test or one-way analysis of variance, followed by a Tukey's post hoc test were employed for the analysis of quantitative variables. P-values <0.05 indicate statistical significance.

## 3 Results

### 3.1 SPDL1 is highly expressed in HCC samples

**Fig. 1:**
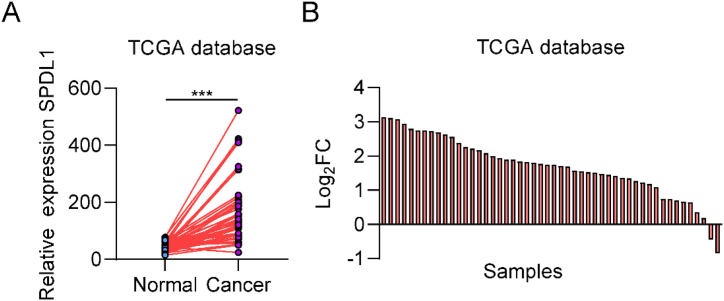
SPDL1 is highly expressed in HCC samples. (A) SPDL1 expression in HCC and adjacent normal tissues based on data obtained from The Cancer Genome Atlas (TCGA) database. (B) The fold change of SPDL1 expression in HCC samples from TCGA as compared to normal tissues. *P < 0.05.

The expression of SPDL1 in patients with HCC was analyzed from TCGA database. The results revealed high expression of SPDL1 in HCC tissues as compared with adjacent normal tissues (Fig. 1A，B).

### 3.2 Knockdown of SPDL1 suppresses HCC cell proliferation and migration

**Fig. 2:**
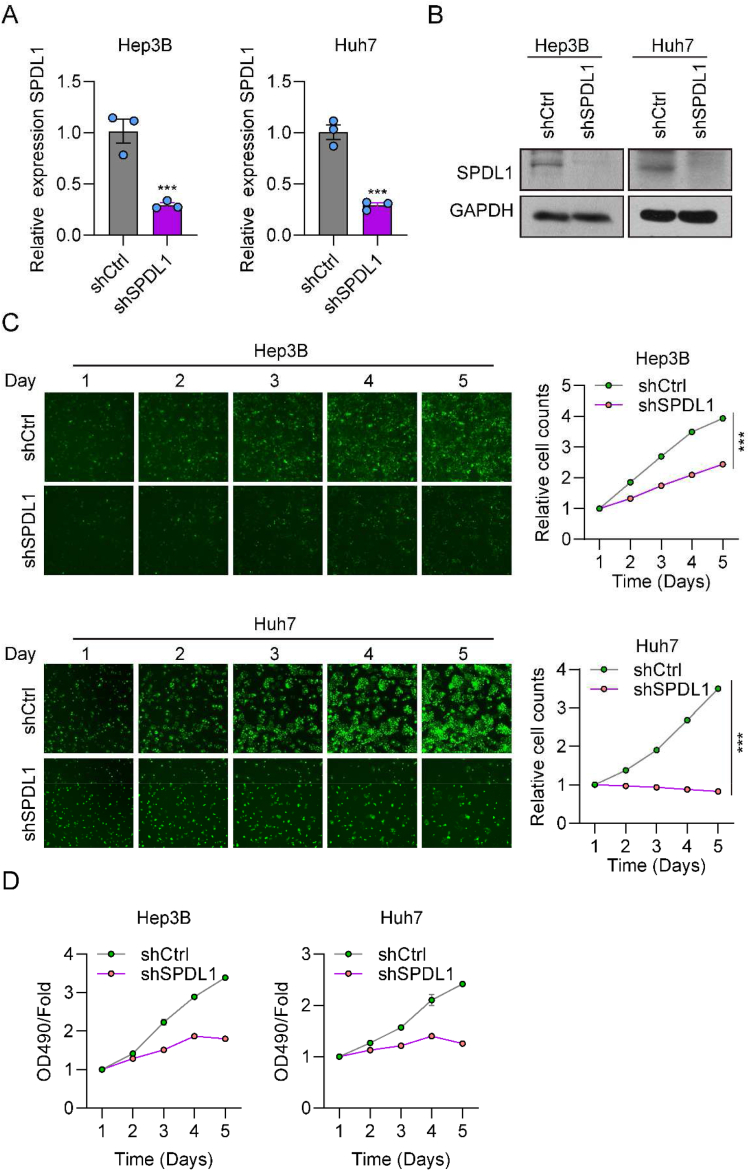
Knockdown of SPDL1 suppresses HCC cell proliferation. (A, B) The mRNA and protein levels of SPDL1 in Hep3B and Huh7 cells were detected by (A) RT-qPCR and (B) western blotting after transfection with shSPDL1 or shCtrl lentivirus. The full and non-adjusted WB results were presented in supplementary material. (C) Representative images and quantification of HCS results for the viability of Hep3B and Huh7 cells transfected with shSPDL1 or shCtrl from day 1 to day 5. (D) MTT assay was used to determine the cell growth rate in HCC cells transfected with shSPDL1 or shCtrl. ***P < 0.001. n = 3 biological replicates.

We sought to evaluate the effect of SPDL1 on the propagation of HCC cells. To this aim, the lentivirus-mediated SPDL1 knockdown assay was performed in Hep3B and Huh7 cells. The knockdown efficiency was also determined by RT-qPCR and immunoblotting (Fig. 2A and B). Furthermore, using the multi-parametric HCS assay, we verified that the proliferation ability of HCC cells was markedly decreased in the shSPDL1 group as compared to the shCtrl group (Fig. 2C). Moreover, the MTT assay yielded consistent results; i.e., silencing of SPDL1 suppressed the viability of Hep3B and Huh7 cells (Fig. 2D). These findings suggested that knockdown of SPDL1 restrained HCC cell proliferation.

### 3.3 Knockdown of SPDL1 inhibits tumor growth *in vivo*

**Fig. 3:**
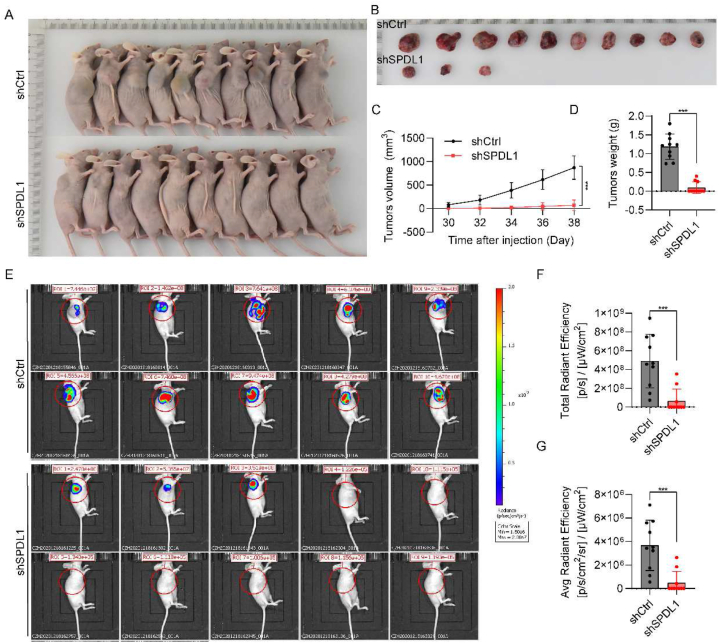
Knockdown of SPDL1 inhibits tumor growth *in vivo*. (A, B) Tumor size of mice between two groups. (C) Growth curves of the tumors between two groups. (D) Tumor weights between two groups. (E) BLI signals of Huh7-SPDL1-shRNA-Red-fLuc cell derived tumor-bearing mice on days 38 post treatment. (F) BLI signal intensity of mice. ***P < 0.001. n = 10 biological replicates.

To further explore the function of SPDL1 *in vivo*, nude mice xenografts were established by subcutaneously injecting Huh7-SPDL1-shRNA-Red-fLuc and Huh7-control-shRNA-Red-fLuc cells. Knockdown of SPDL1 significantly inhibited the initiation and progression of HCC cells (Fig. 3A and B). The tumor volume and weight were decreased in SPDL1 knockdown group comparing with shCtrl group. (Fig. 3C and D). The tumor volume was evaluated by small animal live imaging in the mouse xenografts. SPDL1 depletion suppressed the fluorescence in the xenografts (Fig. 3E–G). Therefore, we demonstrate that SPDL1 knockdown inhibited tumor growth *in vivo*.

### 3.4 Knockdown of SPDL1 promotes cell apoptosis

**Fig. 4:**
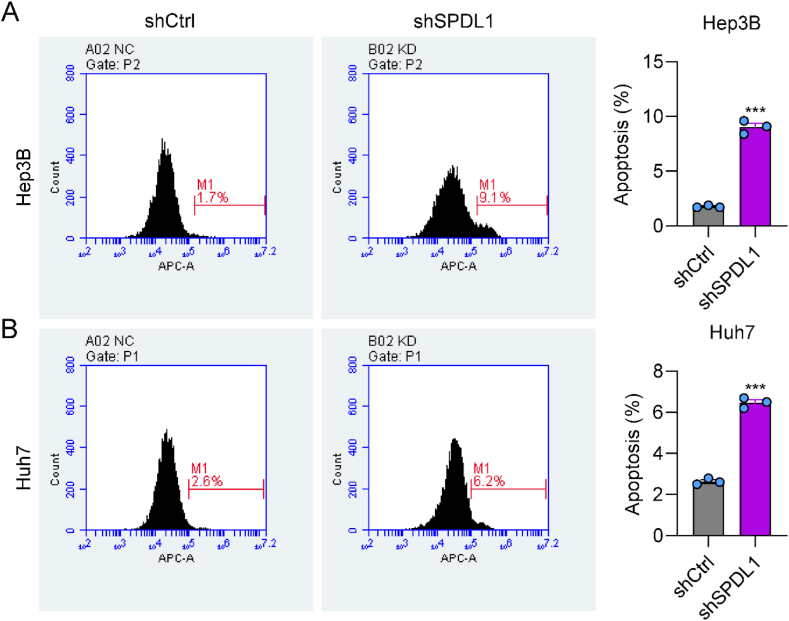
Knockdown of SPDL1 promotes cell apoptosis in (A) Hep3B and (B) Huh7 cells. ***P < 0.001. n = 3 biological replicates.

Next, we examined whether SPDL1 controlled the apoptosis by using Annexin-V-APC staining in the shCtrl and shSPDL1 HCC cells. SPDL1 knockdown increased the rate of apoptosis in both Hep3B and Huh7 cells (Fig. 4A and B), indicating that silencing of SPDL1 enhances apoptosis in HCC cells.

### 3.5 Knockdown of SPDL1 suppresses cell migration

**Fig. 5:**
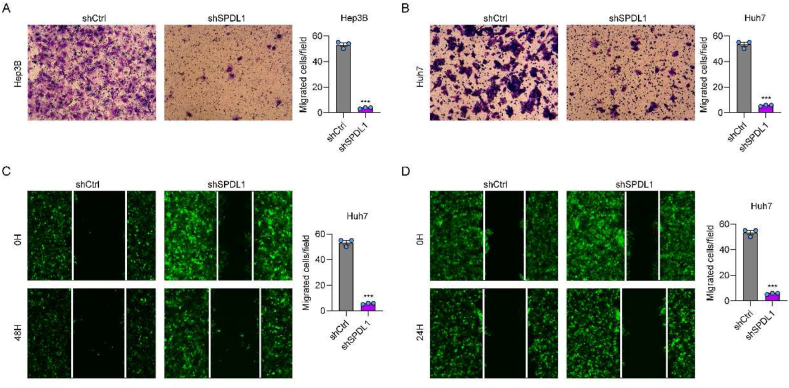
Knockdown of SPDL1 suppresses HCC cell migration. (A, B) The migration capacity of (A) Hep3B and (B) Huh7 cells transfected with shSPDL1 or shCtrl was analyzed by the Transwell assay. (C, D) The migration ability of (C) Hep3B and (D) Huh7 cells transfected with shSPDL1 or shCtrl was analyzed by the wound-healing assay. ***P < 0.001. n = 3 biological replicates.

Transwell assay results showed that SPDL1 knockdown markedly decreased the number of migrated Hep3B and Huh7 cells (Fig. 5A and B). Consistently, the wound-healing assay demonstrated that the migration distance of HCC cells was markedly inhibited by SPDL1 knockdown at 48 h after performing the wound (P < 0.05) (Fig. 5C and D). These findings suggested that knockdown of SPDL1 restrained HCC cell migration.

### 3.6 FNTB is a downstream protein of SPDL1 and its overexpression recovers the proliferative and migratory ability of cells

**Fig. 6:**
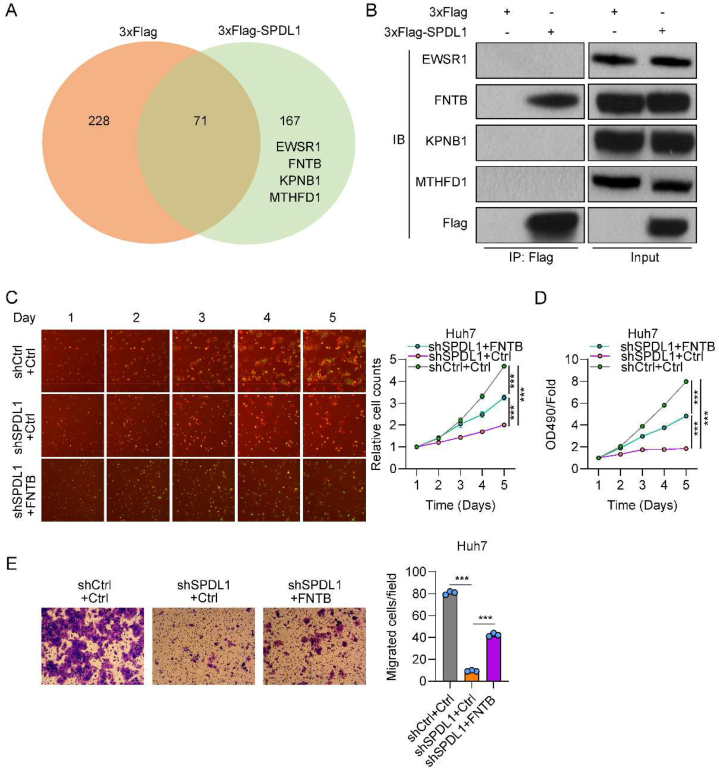
FNTB interacts with SPDL1 and its overexpression recovers the proliferation and migration ability of HCC cells. (A) Venn diagram of proteins recognized by mass spectrometry analysis. (B) Immunoblotting with indicated antibodies was utilized to identify bound proteins in 293 T cells transfected with Flag-SPDL1 or Flag-Con plasmids. The full and non-adjusted WB results were presented in supplementary material. (C) HCS assay was used to analyze the viability of Huh7 cells transfected with shCtrl + Ctrl, shSPDL1+Ctrl, and shSPDL1+FNTB-pEX from days 1–5. (D) Cell growth rate was detected by the MTT assay. (E) The migration ability of cells was analyzed by the Transwell assay. ***P < 0.001. n = 3 biological replicates.

To examine the molecular mechanism underlying the tumor-driving effect of SPDL1, the proteins interacting with SPDL1 were identified with the IP–MS approach. As shown in the Venn diagram, the present MS data analysis identified 167 unique proteins (Fig. 6A). Following the transfection of 293 T cells with Flag-SPDL1 or Flag-Control plasmids and purification using Flag M2 beads, cell lysates were subjected to MS analysis. The MS data analysis recognized several SPDL1-interacting proteins, including EWSR1, FNTB, KPNB1, and MTHFD1. Western blotting assay was further applied to validate the MS data. Among the four proteins, we only detected FNTB in Flag-SPDL1 immunoprecipitate (Fig. 6B). These results indicated the interaction between SPDL1 and FNTB.

To test whether SPDL1 influences the propagation and migration of HCC cells by FNTB, we introduced an overexpression plasmid of FNTB (FNTB-pEX) into HCC cells. HSC assays showed that FNTB-pEX significantly increased Huh7 cell proliferation, which was suppressed by shSPDL1 at days 3, 4, and 5 (Fig. 6C). MTT assay demonstrated that the proliferation capacity of Huh7 cells was markedly increased after overexpression of FNTB compared with that recorded for shSPDL1 cells (Fig. 6D). Furthermore, Transwell assay results showed that FNTB overexpression markedly increased the number of migrated cells, which was formerly inhibited by silencing of SPDL1 (Fig. 6E). These data indicated that FNTB overexpression recovered the proliferation and migration ability of HCC cells suppressed by SPDL1 silencing.

### 3.7 SPDL1 knockdown increases the sensitivity of HCC cells to lenvatinib and sorafenib

**Fig. 7:**
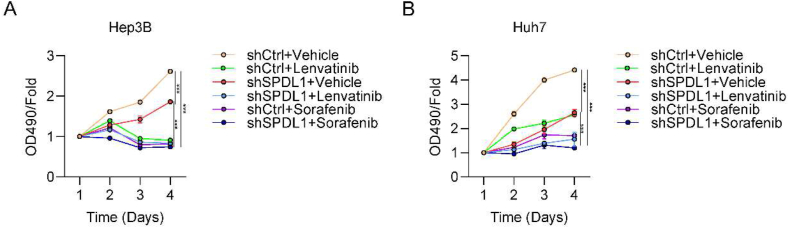
SPDL1 knockdown increases the sensitivity of cells to treatment with lenvatinib and sorafenib. (A and B) shCtrl and shSPDL1 (A) Hep3B and (B) Huh7 cells were treated with vehicle, lenvatinib or sorafenib. The viability of the cells at day 1–4 was measured by MTT assay. *P < 0.05. n = 3 biological replicates.

Previous reports showed that SPDL1-knockdown cells were more sensitive to paclitaxel [11] and cisplatin [14]. Therefore, we also examined whether silencing of SPDL1 could affect the sensitivity of HCC cells to lenvatinib or sorafenib. The MTT assay was used to detect the proliferation rate of Hep3B and Huh7 cells transfected with shSPDL1 or shCtrl in combination with lenvatinib (15 μM) [21] or sorafenib (15 μM) [22] treatment. The results showed that, compared with shCtrl group, shSPDL1 Hep3B and Huh7 cells were more sensitive to the treatment of lenvatinib and sorafenib at different times (Fig. 7A and B). These findings suggested that SPDL1 knockdown increased the sensitivity of Hep3B and Huh7 cells to lenvatinib and sorafenib.

## 4 Discussion

In recent years, several studies have showed that SPDL1 participates in numerous types of cancer in humans, and could be used as a prognostic marker [7,13,14]. Silva et al. reported that Spindly was highly detected in OSCC patients and was associated with increased cellular proliferation [14]. SPDL1 silencing inhibited the proliferation, migration, and invasion of ESCA cells [18]. In the TCGA cohort, SPDL1 was overexpressed in CRC tissue and positively associated with improved survival, chromosome instability phenotype, and various genomic instability (GIN) markers [7]. Kodama et al. [23] also revealed that lower SPDL1 expression levels are markedly related to decreased survival in patients with CRC. Using in-house IHC and bioinformatic exploration of public datasets, Klimaszewska–Wiśniewska et al. [13] found that high levels of SPDL1 protein were linked to an improved outcome in pancreatic ductal adenocarcinoma. However, an in silico study showed that SPDL1 upregulation was significantly associated with worse overall and disease-free survival, as well as advanced tumor phase [12]. Liu et al. [18] also reported upregulation of SPDL1 in ESCA tissues, and the elevated SPDL1 expression was related to age, drinking history, grade, lymph node metastasis, cancer stage, TP53 mutation, and poor prognosis in ESCA. Song et al. [15] displayed that high SPDL1 expression was linked to poor prognosis in lung adenocarcinoma. These studies suggested that the prognostic role of SPDL1 differed between various types of cancer. In this study, we assessed the expression of SPDL1 in HCC samples from TCGA database. SPDL1 was markedly upregulated in HCC samples versus normal tissues. The effect of SPDL1 on HCC cell propagation and migration was also evaluated through lentiviral transduction of shSPDL1. Silencing of SPDL1 reduced cell propagation and migration, and promoted cell apoptosis. Tumor xenograft model assay also demonstrated that knockdown of SPDL1 suppressed tumor growth *in vivo*. These results indicated the oncogenic effect of SPDL1 on HCC progression.

To further investigate the mechanism underlying the effect of SPDL1 on HCC progression, we attempted to identify the downstream proteins of SPDL1 through IP and MS. A total of 167 unique proteins were identified, including EWSR1, FNTB, KPNB1, and MTHFD1. EWSR1 belongs to the FET (also known as TET) family of RNA-binding proteins, which is exposed to aberrations such as rearrangements and involved in the development of diverse benign and malignant tumors [24]. EWSR1 was upregulated in HCC tissues, and this effect was related to the histological grade, pathologic T phase, and death. Hence, EWSR1 belongs to the FET (also known as TET) family of RNA-binding proteins, which is exposed to aberrations such as rearrangements and involved in the development of diverse benign and malignant tumors [25]. FNTB contributes to peripheral T cell homeostasis [26]. Previous research on breast cancer indicated that FNTB promoter polymorphisms were independent prognostic biomarkers, particularly in patients with early TNBC [27]. In HCC, FNTB is a target protein of Ras-converting enzyme 1 (RCE1) and mediates the expression of H-Ras [28]. KPNB1 is a significant nuclear receptor involved in shuttling proteins from the cytoplasm to the nucleus [29]. Several reports revealed that KPNB1 was associated with the progression of various tumors, including glioblastoma [30], breast cancer [31], CRC [32], and non-small cell lung cancer [33]. In HCC, upregulation of KPNB1 expression was found in HCC tissue samples. High expression of KPNB1 increased HCC cell growth via nuclear factor-кB (NF-кB) signaling and was associated with poor survival. These findings indicated KPNB1 as an independent prognostic marker and a new therapeutic target in HCC [34]. MTHFD1 was identified as a novel suppressor of anoikis and facilitated cancer metastasis [35]. In HCC, overexpression of MTHFD1 predicted poorer survival and recurrence [36]. To confirm the involvement of these proteins in SPDL1-induced HCC progression, CO-IP was performed and only FNTB interacted with SPDL1. Introduction of the FNTB-pEX plasmid recovered the proliferation and migration ability of HCC cells with shSPDL1, suggesting that SPDL1 promotes the propagation and migration of HCC cells through interaction with FNTB.

Sorafenib has been approved as the first-line targeted therapy for advanced HCC [37]. Lenvatinib is a multi-kinase inhibitor targeting the vascular endothelial growth factor receptor (VEGFR), fibroblast growth factor receptor (FGFR), and platelet-derived growth factor receptor (PDGFR). In 2018, it was approved as a first-line agent for the treatment of unresectable HCC [38]. Preclinical studies have shown that the sensitivity of sorafenib and lenvatinib was regulated by dysregulation of coding or non-coding RNAs. For example, knockdown of acylphosphatase 1 (ACYP1) reduced lenvatinib resistance in HCC cells [39]. CRISPR/Cas9 screening identified that up-regulated miR-3689a-3p significantly promoted sorafenib sensitivity in HCC cells [40]. Despite the suppression of Spindly was cytotoxic to OSCC cells and strengthened their chemosensitivity to cisplatin [14], the significance of Spindly in sorafenib and lenvatinib sensitivity remains to be determined. Therefore, we evaluated the sensitivity of HCC cells to sorafenib and lenvatinib after SPDL1 knockdown. The results of the MTT assay showed that treatment with lenvatinib or sorafenib in combination with shSPDL1 lowered the viability of Huh7 cells (but not that of Hep3B cells) versus treatment with lenvatinib or sorafenib in shCtrl cells. Our results revealed that the abundance of SPDL1 in HCC cells modulated the toxicity of sorafenib and lenvatinib. The preclinical findings might be helpful to the application of the drugs for the treatment of advanced HCC patients.

Except for the significance of our study, there were also some limitations. For instance, how the interaction between SPDL1 and FNTB protein contributes to HCC proliferation and migration was not clearly illustrated. In addition, the molecular mechanisms, as well as the clinical relevance, of which SPDL1 affected the sensitivity of sorafenib and lenvatinib in HCC cells and patients was not well understood. These questions will be explored in the follow up study.

In conclusion, our study provided the novel evidence that SPDL1 upregulation in HCC contributed to the malignant growth of HCC cells *in vitro* and *in vivo*. Mechanistic insights revealed that the interaction between SPDL1 and FNTB was critical for the proliferation and migration of HCC cells. Importantly, reduction of SPDL1 enhanced the sensitivity of HCC cells to sorafenib and lenvatinib treatment. Our study not only demonstrated SPDL1 as an oncogenic protein in HCC but also illustrated it as a promising biomarker for HCC patients treated with sorafenib and lenvatinib. Therefore, deep studies should be performed in the future to investigate the druggability of SPDL1 and FNTB in HCC.

## Ethics statement

Animal operations were performed according to the National Institutes of Health guidelines, and approved by the Institutional Animal Care and Use Committee of Chinese Academy of Medical Sciences and Peking Union Medical College (NCC2022A481).

## Funding statement

This study was supported by 10.13039/501100004826Beijing Natural Science Foundation (7222151) and 10.13039/501100005150Chinese Academy of Medical Sciences Innovation Fund for Medical Sciences (CIFMS, 2021-I2M-1–066).

## Data availability statement

The datasets used and analyzed during the current study are available from the corresponding author on reasonable request.

## CRediT authorship contribution statement

**Yirui Zhai:** Writing – original draft, Validation, Methodology, Investigation. **Fan Wu:** Writing – review & editing, Validation, Methodology. **Xin Xu:** Writing – review & editing, Validation, Investigation. **Pan Zhao:** Writing – review & editing, Validation, Methodology. **Lingxia Xin:** Writing – review & editing, Validation, Investigation. **Mengyuan Li:** Writing – review & editing, Validation, Investigation. **Yuan Zong:** Writing – review & editing, Validation, Investigation. **Zhuanbo Yang:** Writing – review & editing, Formal analysis, Data curation. **Zhuoran Li:** Writing – review & editing, Formal analysis, Data curation. **Liming Wang:** Writing – review & editing, Supervision, Conceptualization. **Bo Chen:** Writing – review & editing, Supervision, Funding acquisition, Conceptualization.

## Declaration of competing interest

The authors declare that they have no known competing financial interests or personal relationships that could have appeared to influence the work reported in this paper.

## Figures and Tables

**figs1:**